# A *Fucus vesiculosus* extract inhibits estrogen receptor activation and induces cell death in female cancer cell lines

**DOI:** 10.1186/s12906-016-1129-6

**Published:** 2016-05-28

**Authors:** Jianqing Zhang, Jacques E. Riby, Lucia Conde, William E. Grizzle, Xiangqin Cui, Christine F. Skibola

**Affiliations:** Department of Epidemiology, University of Alabama at Birmingham, 1665 University Boulevard, Birmingham, AL 35294 USA; Department of Pathology, University of Alabama at Birmingham School of Medicine, Birmingham, 35294 AL USA; Department of Biostatistics, University of Alabama at Birmingham School of Medicine, Birmingham, 35294 AL USA; The UAB Comprehensive Cancer Center, University of Alabama at Birmingham, 1824 6th Avenue South, Birmingham, 35233 AL USA

**Keywords:** Seaweed, Apoptosis, Autophagy, PI3K signaling, Fucoidan, Anti-estrogenic activity

## Abstract

**Background:**

We previously reported the anti-estrogenic activity of the brown seaweed, *Fucus vesiculosus*. The present study aimed to further investigate its anti-estrogenic modes of action and to assess other potentially biologically relevant anti-tumorigenic effects in estrogen receptor (ER)-dependent and -independent female cancer cell lines.

**Methods:**

The CALUX® assay was used to determine the effect of a *F. vesiculosus* extract (FVE) on activation of the ER. Aromatase enzymatic activity was measured to determine the potential effect of FVE on estradiol (E2) biosynthesis. Transcriptional activity profiling of 248 genes involved in cancer, immunity, hormonal regulation, protein phosphorylation, transcription, metabolism, and cellular structure was conducted using the NanoString nCounter® analysis system in FVE-treated breast, ovarian and endometrial cancer cell lines. The effects of FVE on cell viability, morphology, membrane integrity, mitochondrial toxicity, induction of apoptotic and autophagic markers, and cell signaling were also analyzed.

**Results:**

In co-treatments with 12.5 pM (EC_50_) E2, FVE (2 %) reduced ER activation by 50 %, exhibiting potent ER antagonistic effects. FVE inhibited aromatase activity in an in vitro assay (IC_50_ 2.0 %). ER-dependent and -independent cancer cell lines showed significantly decreased viability that correlated with increasing FVE concentrations and altered morphological features suggestive of apoptosis and autophagy. Expression of genes that were significantly altered by FVE (*p* < 0.05) revealed predominantly apoptotic, autophagic and kinase signaling pathways. FVE also effectively inhibited the phosphorylation of Akt, resulting in reduced mTORC1 activities to stimulate autophagy in cells. Concentration-dependent cleavage of PARP and induction of caspase-3 and -7 activities were observed in MDA-MB-231 cells supporting a role for FVE in the promotion of apoptosis.

**Conclusions:**

Our study provides new insights into the anti-estrogenic activity of *F. vesiculosus*. Moreover, the induction of autophagy and apoptosis on breast, endometrial and ovarian cancer cell lines suggests additional anti-tumorigenic actions of FVE that are independent of ER status in female cancers.

**Electronic supplementary material:**

The online version of this article (doi:10.1186/s12906-016-1129-6) contains supplementary material, which is available to authorized users.

## Background

Brown seaweeds such as *Fucus vesiculosus* are widely consumed by the public due to their potential anti-cancer activities warranting the need for further studies to characterize their biological actions. We previously reported anti-estrogenic properties of *F. vesiculosus* in human and animal studies [1]. Specifically, in a small case study, *F. vesiculosus* administered to pre-menopausal women with endometriosis led to a reduction in circulating estradiol (E2) levels, an increase in the length of the menstrual cycle, and diminished symptoms of hypermenorrhea and dysmenorrhea [1]. Anti-estrogenic action was further demonstrated where dosing with a *F. vesiculosus* extract (FVE) in primary human luteinized granulosa cell cultures led to marked reductions in E2 levels [2]. Rats fed *F. vesiculosus* exhibited increased estrous cycle lengths and reduced serum E2. FVE also exerted inhibitory effects on the binding of E2 to estrogen receptor (ER)α and ERβ [2].

To further explore the anti-estrogenic activity of *F. vesiculosus*, the effects of FVE were tested on the activation of the ER and inhibition of aromatase enzymatic activity. We also investigated other modes of action of FVE biologically relevant to cancer initiation and progression in breast, endometrial and ovarian cancer cell lines. Fucoidan is a major component of *F. vesiculosus* and other brown seaweeds and exhibits anti-tumor and anti-metastatic activities in numerous cancers [3]. Therefore, we included fucoidan purified from *F. vesiculosus* in our in vitro studies to compare its effects with those of FVE.

## Methods

### *F. vesiculosus* aqueous extract preparation

Atlantic *F. vesiculosus* (Maine Coast Sea Vegetables, Inc., Franklin, ME) was ground into a fine powder, mixed with deionized water (5 g into 100 mL) and stirred 2 h at room temperature. The insoluble material was removed by centrifugation; the supernatant (70 mL) was sterilized by filtration through a 0.2 μM filter and stored in 1-mL aliquots at −20 °C. This constitutes the 100 % v/v stock extract. For all experiments, treatment concentrations of the extract are expressed as the % v/v. For example, a 100-fold dilution in cell culture medium is expressed as 1 %. Four aliquots were completely dried under a vacuum using a SpeedVac evaporator overnight yielding 35 mg of solid residue per mL of extract. In total, the 70-mL extract contained 2.45 g of water-soluble material extracted from 5 g of starting plant powder.

### Antibodies and reagents

Antibodies to Akt, phospho-Akt (Ser473), phospho-Akt (Thr308), beclin-1, phospho-Beclin-1 (Ser15), phospho-PI3Kinase p85(Tyr458)/p55 (Tyr199), phospho-4E-BP1 (Thr37/46), p70S6K, phospho-p70S6K(Thr389), LC3B, poly(ADP-ribose) polymerase (PARP), cleaved PARP (D214) and anti-rabbit IgG HRP-linked antibody were purchased from Cell Signaling Technology (Beverly, MA, USA). Mouse monoclonal β-Actin antibody and crude fucoidan from *F. vesiculosus* were obtained from Sigma-Aldrich (St Louis, MO, USA). DMEM (Dulbecco’s Modification of Eagle’s Medium) with 4.5 g/L glucose, L-glutamine and sodium pyruvate, trypsin-EDTA, penicillin-streptomycin-amphotericin B solution (50X), fetal bovine serum (FBS), phosphate buffer solution (PBS), and PBS with Tween 20 (PBST) were purchased from Life Technologies (Waltham, MA, USA).

### Estrogenic activity of FVE measured by a reporter assay

The effect of FVE on E2 signaling was investigated using a chemically activated luciferase reporter (CALUX® assay) for ERα and ERβ. The ER activity reporter, T47D-KBluc cell line, was purchased from ATCC (Manassas, VA). This CALUX® assay cell line is permanently transfected with a plasmid reporter construct expressing luciferase under control of a promoter region containing several repeats of the cognate responsive element for ER. Cells grown in medium depleted of steroids (charcoal filtered FBS (5 %) in DMEM without Phenol-red) for 7 days to minimize background activity were seeded in opaque 96-well plates and allowed to attach overnight. Medium containing 0 to 25 pM E2 as the calibration standard, or FVE (0 to 2 %) either alone or in co-treatments with 12.5 pM E2, was added to the wells in triplicates. Fucoidan was also tested in this assay at a range of concentrations (0 to 0.50 mg/mL). After a 24-h incubation, cells were lysed and luciferase activity was measured with a microplate luminometer using the Promega Flash Luciferase Assay kit (Madison, WI). A clear 96-well plate was seeded and treated identically and was used to normalize the luminescence raw data for possible cell number variations (measured with the MTT assay) due to the 24-h exposure to the treatments. Estrogenic activity was expressed as pM E2 equivalents.

### Effects of FVE on aromatase activity

Aromatase activity was measured using the CYP19/MFC High Throughput Inhibitor Screening Kit (Discovery Labware, Inc. Wolburn, MA) according to the manufacturer’s instructions. Briefly, recombinant human CYP19 enzymatic hydrolysis of the fluorescent substrate, 7-methoxy-4-trifluoromethyl coumarin, was used to measure aromatase activity in vitro in a 96-well plate. Ketoconazole was used as the positive marker of aromatase inhibition. FVE was tested in this assay at a 0 to 2 % range of concentrations.

### Cell lines and culture conditions

Human MDA-MB-231 and MCF-7 breast cancer cells were kindly provided by Dr. Samant (University of Alabama, Birmingham, AL) and the breast carcinoma (T47D), ovarian carcinoma (OVCAR-3 and Caov-3), uterine endometrium carcinoma (HEC-1-B, RL95-2 and AN3CA) and uterine sarcoma (MES-SA) cell lines were obtained from ATCC. The MDA-MB-231/GFP-LC3 stable cell line was a gift from Dr. Xu [4] (University of Alabama, Birmingham, AL). Cell lines were maintained in their respective ATCC recommended media at 37 °C, in 100 % humidity and 5 % CO_2_.

### Effects of FVE on cell viability

The effects of FVE on the viability of all nine cell lines were analyzed using the MTT assay (Promega Corp. Madison, WI). Specifically, cells were seeded at 10^5^ per well in 12-well plates and after overnight attachment, FVE (0 to 2 %) was added to the medium in triplicate wells. After 72 h, MTT dye was added to the medium, incubated one hr at 37 °C. The formazan crystals were dissolved in Solubilization Solution/Stop Mix and the OD 570 nm was used as a measurement of viable cell number.

### Toxicity assessment

The Mitochondrial ToxGlo™ Assay (Life Technologies, Carlsbad, CA) was used to assess cell toxicity, which relies on biomarkers associated with changes in cell membrane integrity and cellular ATP levels. Cell membrane permeability in necrotic cells is indicated by the presence of specific protease activity, using the fluorescent proteolytic product of the peptide substrate (bis-AAF-R110). Digitonin is a positive marker for cellular membrane permeabilization. Carbonyl cyanide *m*-chlorophenyl hydrazone (CCCP) is a positive marker of mitochondrial toxicity.

### Gene expression profiling

As an initial screening tool, transcript levels were measured (nCounter® Analysis System, (NanoString Technologies, Seattle, WA) in a panel of 248 genes involved in cancer, immunity, hormonal regulation, protein phosphorylation, transcription, metabolism, and cellular structure (Additional file 1: Table S1) in a subset of 6 cell lines (MCF-7, T47D, MDA-MB-231, RL95, HEC-1-B, RL95-2 and OVCAR-3). Cells were treated with FVE (0, 0.25 and 1.0 %, up to 4 h) and mRNA transcript levels were measured using total RNA that was purified using the Qiagen RNeasy Mini Kit and quantified using a BioTek Take-3 UV spectrophotometer. The geometric mean of six housekeeping genes (*TUBB, G6DP, GAPDH, POLR2A, RPL19* and *TBP*) was used as the reference for normalization of measured mRNA levels. To filter out genes expressed at low background levels, we removed those with expression levels below 4 in all samples after normalization (70 counts in the raw data). For the remaining genes, a two-way ANOVA model with cell line effect and treatment dose effect was fitted to the normalized and logarithm-transformed expression data for each gene. The permutation-based F-test was used to identify genes differentially expressed between treatment doses. Due to the screening characteristics, we used a nominal p-value of 0.05 as significance threshold. Analyses were conducted in R, version 3.0.0.

### Assessment of FVE on apoptosis and autophagy with pan-caspase and autophagy inhibitors

The relative contribution of apoptosis and autophagy to the reduced viability of the cancer cells exposed to FVE was examined using co-treatment with specific inhibitors. The pan-caspase inhibitor, Z-VAD (OMe)-FMK (VAD, 60 μM) and the autophagy inhibitor, 3-methyladenine (3-MA, 8 mM; Cayman Chemicals, Ann Arbor, MI) were used in co-treatments with 1 % FVE -for 72 h.

### Caspase activity assay

Caspase activity was determined using the EnzChek Caspase-3 Assay Kit #1 (Molecular Probes, Grand Island, NY). Briefly, estrogen-dependent MCF-7 and estrogen-independent MDA-MB-231 breast cancer cells were incubated 2 h with 1 % FVE. Cells were collected after trypsin digestion, washed in PBS, and lysed in 1x lysis buffer by freeze and thaw. After centrifugation (5,000 x g, 5 min), the supernatant containing 100 μg protein was incubated with substrate (Z-DEVD-AMC, 25 °C, 30 min) or pre-incubated with caspase-3/7 inhibitor (Ac-DEVD-CHO) (10 min) before addition of substrate (Z-DEVD-AMC). Released AMC fluorescence was measured with a microplate reader (Synergy-H1) at excitation/emission 342/441 nm and the net change of fluorescence was calculated by subtraction of the fluorescence of the sample pre-incubated with the inhibitor. Caspase-3/7 activities were determined by direct comparison to the level in the untreated control cells. For caspase inhibitor treatments, cells were pretreated with a synthetic cell-permeable pan-caspase inhibitor (Z-VAD (OMe)-FMK, 10 mM, 30 min) before the addition of 1 % FVE and incubated for an additional 2 h.

### Fluorescence microscopy

MDA-MB-231 cells/GFP-LC3 were grown to 60 % confluence in six-well plates with cover slides in DMEM supplemented with 10 % FBS. Cells were treated with FVE (0 to 2 %, 16 h) and fixed in 4 % neutral paraformaldehyde in PBS for 15 min at room temperature and washed 3 times in PBS. Slides were mounted with antifade medium Dapi Fluoromount-G (Southern Biotech, Birmingham, AL) and the GFP-LC3 staining was observed using a fluorescence microscope.

### Western blots

Immunoblots were used to study the effects of FVE on phosphorylation and activation of kinases in the PI3K-Akt-mTOR pathway. Cells grown in 6-well plates were treated with FVE (0 to 2 %, up to 8 h), then lysed in RIPA buffer supplemented with a cocktail of protease and phosphatase inhibitors. After the addition of 2X Laemmli loading solution containing 10 % β-mercaptoethanol, cell lysates were sonicated 5 s and denatured at 100 °C for 5 min. SDS-Page Gels (Mini-Protean TGX Precast Gels, Any kD, BioRad, Hercules, CA) were loaded with 30 μg of sample per lane, run at 200 V for 40 min and transferred to PVDF membranes using the rapid semi-dry Trans-Blot Turbo Transfer System (BioRad, Hercules, CA). Blots were blocked in 5 % non-fat dry milk at 37 °C for 30 min followed by overnight incubation at 8 °C with primary antibodies in blocking buffer. Secondary antibodies conjugated to HRP were imaged by enhanced chemoluminescence on a ChemiDoc MP imaging system (BioRad) and analyzed using BioRad’s Image Lab software. Blots were re-probed with different antibodies after treatment with Restore™ Plus Western Blot Stripping Buffer (Thermo Fisher Scientific, Rockford, IL).

### Statistical analyses

Experimental data were analyzed by the Student’s *t*-test. Data are shown as means ± S. E. of values obtained from triplicate or quadruplicate experiments. A cut-off value of *p* < 0.05 was used to indicate statistical significance.

## Results and discussion

### FVE inhibits ER activation and aromatase activity

**Fig. 1:**
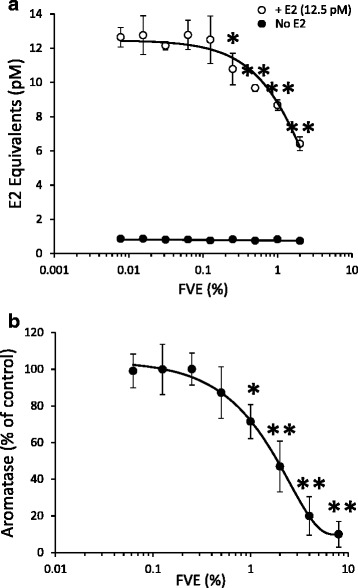
Anti-estrogenic effects of FVE through inhibition of estrogen receptor (ER) activation and aromatase activity. **a** In co-treatment with E2 (EC50, 12 pM), FVE inhibited ER activation with an IC50 of 2 %, but exerted no effect on ER activity alone. **b** FVE inhibited aromatase enzymatic activity in vitro with an IC50 of 2 %. *p < 0.05; **p < 0.001

We previously reported the anti-estrogenic activity of *F. vesiculosus*, and that one mode of action was competitive inhibition of E2 binding to the ER [1, 2]. However, the effect of the extract upon ER activation was never tested. Here, using the Calux assay, we found that in cells co-treated with E2 at 12.5 pM (EC_50_), FVE significantly reduced the activation of the luciferase reporter up to 50 % (*p* < 0.001) (Fig. 1a), suggesting that FVE is a potent antagonist of ER activation by E2. Moreover, in vitro enzymatic activity of aromatase was inhibited by FVE with an IC_50_ of 2 % (Fig. 1b). However, fucoidan did not alter ER activation or aromatase activity (data not shown) even at concentrations in excess of the 10 % of dry weight reported for *F. vesiculosus* [5]. Inhibition of aromatase enzymatic activity by FVE provides a potential mechanism for the decreased serum E2 levels that we previously reported following consumption of *F. vesiculosus* [1]*.* The dual actions of an antagonistic effect on the activation of the ER by circulating E2, together with reduced levels of E2 synthesis through aromatase inhibition, suggests that *F. vesiculosus* may play a protective role in the initiation and progression of estrogen-dependent cancers.

### Reduced viability in FVE-treated cancer cell lines

**Fig. 2:**
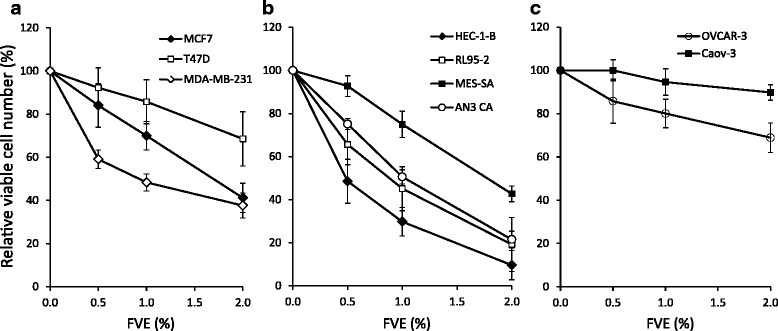
Cell viability as measured by the MTT assay in (**a**) breast (MCF-7, T47D, MDA-MB-231), (**b**) uterine (HEC-1-B, RL95-2, MES-SA, AN3-CA), and (**c**) ovarian (OVCAR-3, Caov-3) cancer cell lines

As measured by the MTT assay of mitochondrial activity, treatment with FVE showed reduced cell viability after a 3-day period in most cancer cell lines tested, in a dose-dependent fashion, though sensitivity was highly variable between cell lines (Fig. 2a-c). FVE exerted the greatest reduction in cell viability in the HEC-1-B and RL95-2 endometrial cells (Fig. 2b); whereas, there was no significant effect on cell viability in the Caov-3 ovarian carcinoma cells (Fig. 2c). The cell line-specific sensitivity suggests that FVE is not toxic at the concentration range in our experiments, but instead induces cell death through modulated pathways. These observations also support our previous reports of the non-toxic effects of *F. vesiculosus* in vivo [1, 2]. The toxicity of FVE was further studied in a dual assay of mitochondrial production of ATP and of cell membrane permeability. Treatment with FVE at the same concentrations (up to 2 %) shown to reduce cell viability in MCF-7 cells did not produce toxic effects when compared to the effects of the positive markers for mitochondrial toxicity (CCCP) and for membrane permeabilization (digitonin), indicating that there was no necrotic effect due to primary toxicity (Additional file 1: Figure S1 A-C). Results were similar for all cell lines tested (data not shown). In the course of the cell proliferation experiments, we observed morphological changes within the cells, featuring predominantly large cytoplasmic vesicles after 72 h of treatment with 1 % FVE. Additional file 1: Figure S2 shows typical morphology alterations in six representative cell lines.

### FVE treatment alters expression of genes involved in apoptosis, autophagy and cell signaling

Gene expression profiling studies revealed that breast, endometrial and ovarian cells treated with FVE compared to untreated controls exhibited differentially altered transcript levels (*p* < 0.05) in genes primarily involved in apoptosis (*APAF1, CASP6, FANCG, MED1, XIAP*), autophagy (*ATG10, GABARAP*) and in genes in signaling pathways modulated by protein kinases (*BRAF, MAP3K14, PIK3R4, PRKAA1, PRKACB, PRKAR1A, PRKAR2A)* (Additional file 1: Figure S3). These results suggested that the reduced cell viability by FVE was through induction of apoptosis and/or autophagy warranting further investigation of these mechanisms. Morphological observations of the cancer cells exposed to FVE also supported initiation of cell death by apoptosis and/or autophagy (Additional file 1: Figure S3).

### FVE-induced reduction of cancer cell viability is attenuated by specific inhibitors of apoptosis and autophagy

**Fig. 3:**
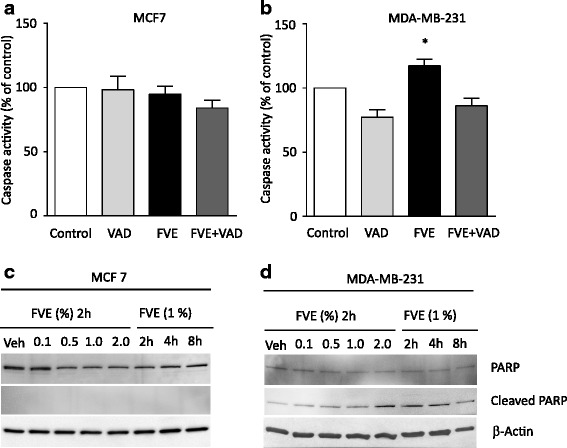
Effects of FVE on apoptosis in (**a**) MCF-7 cells and (**b**) MDA-MB-231 cells with the pan-caspase inhibitor, VAD. **c** Effects of FVE on PARP cleavage in MCF-7 and (**d**) MDA-MB-231 cells. The antibody does not recognize full length PARP1 or other PARP isoforms. *p <0.05; **p < 0.01 compared to control

The contributions of apoptosis and autophagy to the reduced viability of the cancer cells exposed to FVE were examined using co-treatments with VAD, the pan-caspase inhibitor, and 3-MA, the autophagy inhibitor, in MCF-7, MDA-MB-231, HEC-1-B, MES-SA, AN3-CA, OVCAR-3 and Caov-3 cells. We found that co-treatment with VAD reversed the actions of FVE on cell viability in MDA-MB-231, AN3-CA and OVCAR cells (Additional file 1: Figure S4). To further explore the contribution of apoptosis in the reduction of cell viability by FVE, we measured caspase activity in MCF-7 and MDA-MB-231 cells (Fig. 3a and b) as representative of VAD-unresponsive and -responsive cell lines, respectively. FVE did not affect caspase activity in MCF-7 cells (Fig. 3a), which is consistent with the lack of caspase-3 expression in MCF-7 cells [6]. However, caspase activity was increased in FVE-treated MDA-MB-231 cells, and the addition of VAD reduced this activity (Fig. 3b). PARP cleavage by caspase-3 serves as one hallmark of apoptosis and caspase activation [7]. Thus, we used Western blotting with an antibody specific for cleaved PARP which showed no apparent PARP cleavage in MCF-7 cells (Fig. 3c), but increased PARP cleavage in FVE- treated MDA-MB-231 cells (Fig. 3d) in a concentration-dependent manner (Additional file 1: Figure S5). These studies suggest that apoptosis is not the primary mechanism in the cell killing effect of FVE on MCF-7 cells, but apoptosis does play a role in cell death in MDA-MB-231 cells.

**Fig. 4:**
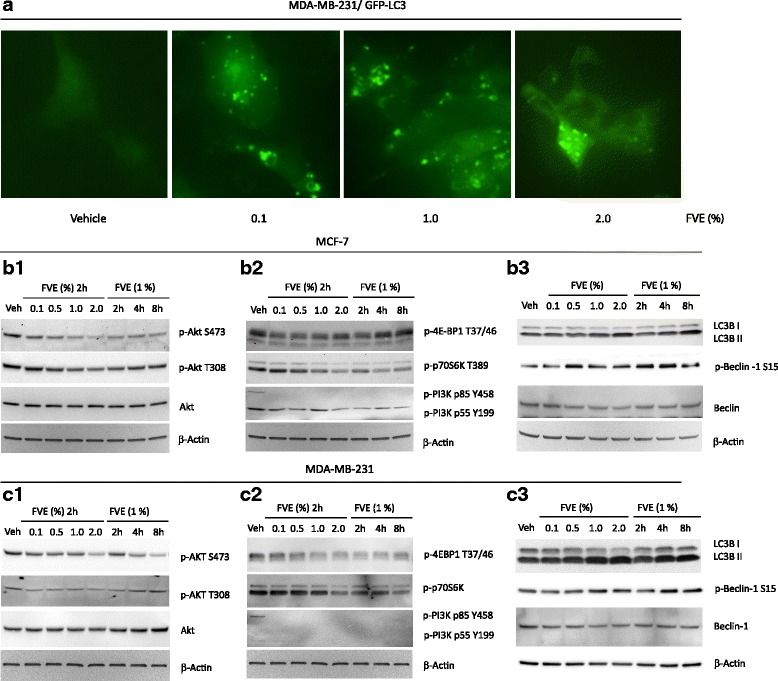
FVE-induced autophagy. **a** Accumulation of GFP-LC3 punctate vesicles in MDA-MB-231 cells. (b1 and c1) FVE reduced Akt phosphorylation at Ser473 and Thr308 in MCF-7 and MDA-MB-231 cells. (b2 and c2) FVE decreased PI3K, 4-EB-P1 and p70S6K phosphorylation in MCF-7 and MDA-MB-231 cells. (b3 and c3) FVE promoted accumulation of phospho-Beclin 1 and LC3B II in MCF-7 and MDA-MB-231 cells

When cells were co-treated with FVE and 3-MA, the effects of FVE on cell viability were diminished in all cell lines tested, except in Caov-3 cells which were not responsive to FVE, indicating that autophagy plays a predominant role in FVE-induced cell death (Additional file 1: Figure S4). These results are consistent with the report by Cheng et. al. that showed the reversal of autophagy by 3-MA in MCF-7 and MDA-MB-231 cell lines [8].

### FVE induces autophagy in MDA-MB-231 cells

FVE-induced autophagy was further demonstrated using MDA-MB-231/GFP-light-chain 3 (LC3), a stable cell line expressing green fluorescent protein (GFP)-tagged LC3 (Fig. 4a). During the autophagic process, LC3 is concentrated in autophagic vesicles (autophagosomes) forming cytosolic punctate fluorescence, which serves as an indicator of autophagy [9, 10]. Overnight treatment with FVE at concentrations as low as 0.1 % led to discrete vesicular structures (GFP puncta, Fig. 4a).

### Inhibition of PI3K/Akt/mTOR signaling by FVE

It is well established that PI3K is associated with numerous cellular processes including cell growth, proliferation, differentiation, motility, survival and intracellular trafficking. Many of these functions relate to the ability of class I PI3-kinases to activate Akt in the PI3K/Akt/mTOR pathway [11]. mTOR is a critical effector of PI3K signaling in many human cancers [12], and disruption of the pathway results in apoptotic cell death [13, 14]. Moreover, autophagy is tightly controlled by the mTOR-dependent signal transduction pathway, and inhibition of mTOR activity promotes autophagy [15, 16].

To further explore these mechanisms, we studied the effects of FVE on phosphorylation and activation of kinases in the PI3K-Akt-mTOR pathway in MCF-7 and MDA-MB-231 cells. Western blot data are presented in Fig. 4 as representative ER-positive and ER-negative cell lines and their respective quantifications are shown in Additional file 1: Figure S6 and S7. FVE significantly reduced Akt phosphorylation at Ser473 and Thr308, without changing the levels of total Akt in MCF-7 (Fig. 4b1) and MDA-MB-231 (Fig. 4c1) cells. The effects were dose-dependent and were achieved in less than 2 h. Longer treatments (4 and 8 h) showed no further decrease of p-Akt-Ser/Thr in MCF-7 cells, but in MDA-MB-231 cells at 8 h, there was a more marked decrease of p-Akt-Ser473. Consistent with the inactivation of Akt, the phosphorylation of the p85 regulatory subunit of PI3K was decreased with FVE treatment in MCF-7 and MDA-MB-231 cells (Fig. 4b2 and c2). We also observed that FVE-induced inhibition of Akt phosphorylation resulted in decreased mTOR complex activity as evidenced by a significant reduction in levels of the mTORC1 effectors, p-4E-BP1 and p-p70S6K (Fig. 4b2 and c2). The effect of FVE on the reduction of p-Akt-Ser/Thr was observed in T47D, OVCAR-3, HEC-1-B, RL95-2, AN3-CA and MES-SA cell lines (data not shown), but not in Caov-3 cells where there were no detectable p-Akt-Ser/Thr levels. This is consistent with the lack of Akt activity in Caov-3 cells [17] and may explain the ineffectiveness of FVE on reducing Caov-3 cell viability. These results suggest that FVE is a mediator of autophagic cell death through reduced phosphorylation of key components of the PI3K/Akt/mTOR pathway.

### FVE promotes conversion of LC3B-I to LC3B-II and increases phosphor-Beclin 1

To further explore the effects of FVE treatment on autophagy, we examined the autophagic marker, LC3B. We observed significant conversion of LC3B-I to the smaller form, LC3B-II (Fig. 4b3, c3), which was concentration- and time-dependent. LC3B-I conversion to LC3B-II through ubiquitination allows LC3B association with autophagic vesicles. The presence of LC3B in autophagosomes, and the conversion of LC3B-I to LC3B-II are used as indicators of autophagy [18–20]. Increased levels of phospho-Beclin 1 also were seen after treatment with FVE (Fig. 4b3, c3). The balance between apoptosis and autophagy is regulated by Beclin 1 and is dependent on the dose of the stimulus [21, 22]. The higher levels of phospho-Beclin 1 induced by FVE further suggest that aberrant autophagy is the predominant process involved in its action in promoting cell death.

### Phosphorylation of Akt is not effected by fucoidan

In contrast to what we observed with FVE (Fig. 4b1, c1), fucoidan did not reduce p-Akt at Ser473 or Thr308 levels (Additional file 1: Figure S8). The lack of inactivation of Akt and no apparent effects on proliferation suggest that fucoidan is not the active compound responsible for the inhibition of PI3K/Akt/mTOR signaling by FVE in the cell lines tested.

## Conclusions

Our results provide further evidence of the anti-estrogenic activity exerted by FVE that involves inhibition of ER activation by E2, and inhibition of E2 synthesis. This activity suggests that FVE may provide a beneficial protective effect against estrogen-dependent breast, endometrial and ovarian cancers. Moreover, the reduced viability of ER-positive and -negative cancer cell lines treated with FVE through inactivation of the PI3K/Akt/mTOR pathway suggests that FVE may be useful in the treatment of some breast, endometrial and ovarian cancers regardless of ER status. MDA-MB-231 breast, HEC-1B endometrial and OVCAR-3 ovarian cells do not express the ER, but were sensitive to FVE; HEC-1B endometrial carcinoma cells were the most sensitive to FVE. Endometrial cancers are often aggressive, and therapeutic options are limited and sometimes ineffective. Therefore, the identification of new therapeutic agents is highly desirable. Studies to further characterize the chemoprotective and anti-tumorigenic actions of FVE are currently underway.

## Abbreviations

ATCC, American Type Culture Collection; ATP, adenosine triphosphate; CCCP, carbonyl cyanide *m*-chlorophenyl hydrazine; E2, estradiol; EC_50,_ half maximal effective concentration; ER, estrogen receptor; FVE, *Fucus vesiculosus* extract; FBS, fetal bovine serum; GFP, green fluorescent protein; LC3, light-chain 3; 3-MA, 3-methyladenine; MTT, 3-(4,5-dimethylthiazol-2-yl)-2,5-diphenyltetrazolium bromide; PARP, poly(ADP-ribose) polymerase; PBS, phosphate buffer solution; PBST, phosphate buffer solution with Tween 20; VAD, Benzyloxycarbonyl-Val-Ala-Asp (OMe) fluoromethylketone (Z-VAD.FMK).

## Additional file

Additional file 1: Table S1. 248 genes analyzed for expression profiling (Nanostring™ nCounter®) and 6 housekeeping reference genes. **Figure S1.** Assays of toxicity. (A) FVE effects on membrane permeability and mitochondrial ATP. (B) Digitonin used as positive control for primary necrosis. (C) CCCP used as positive control for mitochondrial toxicity. **Figure S2.** Morphological alterations. (A) FVE-untreated and (B) -treated cells with 1.0 % FVE, 48 hr. **Figure S3.** Heatmap of differential mRNA expression following FVE treatment at 0.25 % and 1.0 % (4 hr) in MCF-7, T47D, MDA-MB-231, HEC-1-B, RL95-2 and OVCAR-3 cell lines; significance level, *p* <0.05. **Figure S4.** Treatment of MCF-7, MDA-MB-231, HEC-1-B, MES-SA, AN3-CA, OVCAR-3 and Caov-3 cells with apoptosis (VAD) and autophagy (3MA) inhibitors; *indicates significant difference with FVE without inhibitor (*p* <0.05). **Figure S5.** FVE-induced apoptosis via caspase3/7-mediated PARP cleavage in MDA-MB-231 cells; **p* <0.05, ***p* <0.01 compared to controls. **Figure S6.** FVE down-regulates PI3K/Akt/mTOR signaling in MCF-7 cells. (A) FVE reduced Akt phosphorylation at Ser473 and Thr308, (B) decreased PI3K, 4-EB-P1 and p70S6K phosphorylation, and (C) promoted accumulation of phospho-Beclin-1 and LC3B II. Data are from >3 independent Western blots normalized by β-actin levels; **p* <0.05, ***p* <0.01 compared to controls. **Figure S7.** FVE down-regulates PI3K/Akt/mTOR signaling in MDA-MB-231 cells. (A) FVE reduced Akt phosphorylation at Ser473 and Thr308, (B) decreased PI3K, 4-EB-P1 and p70S6K phosphorylation, and (C) promoted phospho-Beclin-1 and LC3B II accumulation. Data are from >3 independent Western blots normalized by β-actin levels; **p* <0.05, ***p* < 0.01 compared to controls. **Figure S8.** Fucoidan up-regulates phosphor-Akt. (A) Fucoidan increased Akt phosphorylation at Ser473 in MCF-7 cells in a concentration-dependent manner; no change in Akt phosphorylation at Thr308. (B) Fucoidan increased Akt phosphorylation at Ser473 in MDA-MB-231 cells in a concentration- and time-dependent manner; no changes observed in Akt phosphorylation at Thr308. (PDF 11350 kb)

## References

[1] Skibola CF (2004). The effect of Fucus vesiculosus, an edible brown seaweed, upon menstrual cycle length and hormonal status in three pre-menopausal women: a case report. BMC Complement Altern Med.

[2] Skibola CF, Curry JD, VandeVoort C, Conley A, Smith MT (2005). Brown kelp modulates endocrine hormones in female Sprague–Dawley rats and in human luteinized granulosa cells. J Nutr.

[3] Senthilkumar K, Manivasagan P, Venkatesan J, Kim SK (2013). Brown seaweed fucoidan: biological activity and apoptosis, growth signaling mechanism in cancer. Int J Biol Macromol.

[4] Xu ZX, Liang J, Haridas V, Gaikwad A, Connolly FP, Mills GB, Gutterman JU (2007). A plant triterpenoid, avicin D, induces autophagy by activation of AMP-activated protein kinase. Cell Death Differ.

[5] Fitton JH (2011). Therapies from fucoidan; multifunctional marine polymers. Mar Drugs.

[6] Janicke RU (2009). MCF-7 breast carcinoma cells do not express caspase-3. Breast Cancer Res Treat.

[7] Lazebnik YA, Kaufmann SH, Desnoyers S, Poirier GG, Earnshaw WC (1994). Cleavage of poly(ADP-ribose) polymerase by a proteinase with properties like ICE. Nature.

[8] Cheng SM, Chang YC, Liu CY, Lee JY, Chan HH, Kuo CW, Lin KY, Tsai SL, Chen SH, Li CF (2015). YM155 down-regulates survivin and XIAP, modulates autophagy and induces autophagy-dependent DNA damage in breast cancer cells. Br J Pharmacol.

[9] Kabeya Y, Mizushima N, Ueno T, Yamamoto A, Kirisako T, Noda T, Kominami E, Ohsumi Y, Yoshimori T (2000). LC3, a mammalian homologue of yeast Apg8p, is localized in autophagosome membranes after processing. EMBO J.

[10] Mizushima N, Yamamoto A, Matsui M, Yoshimori T, Ohsumi Y (2004). In vivo analysis of autophagy in response to nutrient starvation using transgenic mice expressing a fluorescent autophagosome marker. Mol Biol Cell.

[11] Franke TF, Kaplan DR, Cantley LC, Toker A (1997). Direct regulation of the Akt proto-oncogene product by phosphatidylinositol-3,4-bisphosphate. Science.

[12] Easton JB, Houghton PJ (2006). mTOR and cancer therapy. Oncogene.

[13] Vivanco I, Sawyers CL (2002). The phosphatidylinositol 3-Kinase AKT pathway in human cancer. Nat Rev Cancer.

[14] Shaw RJ, Cantley LC (2006). Ras, PI(3)K and mTOR signalling controls tumour cell growth. Nature.

[15] Ravikumar B, Vacher C, Berger Z, Davies JE, Luo S, Oroz LG, Scaravilli F, Easton DF, Duden R, O'Kane CJ (2004). Inhibition of mTOR induces autophagy and reduces toxicity of polyglutamine expansions in fly and mouse models of Huntington disease. Nat Genet.

[16] Meijer AJ, Codogno P (2009). Autophagy: regulation and role in disease. Crit Rev Clin Lab Sci.

[17] Tang HJ, Jin X, Wang S, Yang D, Cao Y, Chen J, Gossett DR, Lin J (2006). A small molecule compound inhibits AKT pathway in ovarian cancer cell lines. Gynecol Oncol.

[18] Kouroku Y, Fujita E, Tanida I, Ueno T, Isoai A, Kumagai H, Ogawa S, Kaufman RJ, Kominami E, Momoi T (2007). ER stress (PERK/eIF2alpha phosphorylation) mediates the polyglutamine-induced LC3 conversion, an essential step for autophagy formation. Cell Death Differ.

[19] Wang Y, Wang JW, Xiao X, Shan Y, Xue B, Jiang G, He Q, Chen J, Xu HG, Zhao RX (2013). Piperlongumine induces autophagy by targeting p38 signaling. Cell Death Dis.

[20] Kirisako T, Ichimura Y, Okada H, Kabeya Y, Mizushima N, Yoshimori T, Ohsumi M, Takao T, Noda T, Ohsumi Y (2000). The reversible modification regulates the membrane-binding state of Apg8/Aut7 essential for autophagy and the cytoplasm to vacuole targeting pathway. J Cell Biol.

[21] Gurpinar E, Grizzle WE, Shacka JJ, Mader BJ, Li N, Piazza NA, Russo S, Keeton AB, Piazza GA (2013). A novel sulindac derivative inhibits lung adenocarcinoma cell growth through suppression of Akt/mTOR signaling and induction of autophagy. Mol Cancer Ther.

[22] Kang R, Zeh HJ, Lotze MT, Tang D (2011). The Beclin 1 network regulates autophagy and apoptosis. Cell Death Differ.

