# Supplementary material for: A Fucus vesiculosus extract inhibits estrogen receptor activation and induces cell death in female cancer cell lines
Source: BMC Complement Altern Med. 2016 May 28;16:151. doi: 10.1186/s12906-016-1129-6 (PMC4884380; doi:10.1186/s12906-016-1129-6)
Supplement: Additional file 1: Table S1. — 248 genes analyzed for expression profiling (Nanostring™ nCounter®) and 6 housekeeping reference genes. Figure S1. Assays of toxicity. (A) FVE effects on membrane permeability and mitochondrial ATP. (B) Digitonin used as positive control for primary necrosis. (C) CCCP used as positive control for mitochondrial toxicity. Figure S2. Morphological alterations. (A) FVE-untreated and (B) -treated cells with 1.0 % FVE, 48 hr. Figure S3. Heatmap of differential mRNA expression following FVE treatment at 0.25 % and 1.0 % (4 hr) in MCF-7, T47D, MDA-MB-231, HEC-1-B, RL95-2 and OVCAR-3 cell lines; significance level, p <0.05. Figure S4. Treatment of MCF-7, MDA-MB-231, HEC-1-B, MES-SA, AN3-CA, OVCAR-3 and Caov-3 cells with apoptosis (VAD) and autophagy (3MA) inhibitors; *indicates significant difference with FVE without inhibitor (p <0.05). Figure S5. FVE-induced apoptosis via caspase3/7-mediated PARP cleavage in MDA-MB-231 cells; *p <0.05, **p <0.01 compared to controls. Figure S6. FVE down-regulates PI3K/Akt/mTOR signaling in MCF-7 cells. (A) FVE reduced Akt phosphorylation at Ser473 and Thr308, (B) decreased PI3K, 4-EB-P1 and p70S6K phosphorylation, and (C) promoted accumulation of phospho-Beclin-1 and LC3B II. Data are from >3 independent Western blots normalized by β-actin levels; *p <0.05, **p <0.01 compared to controls. Figure S7. FVE down-regulates PI3K/Akt/mTOR signaling in MDA-MB-231 cells. (A) FVE reduced Akt phosphorylation at Ser473 and Thr308, (B) decreased PI3K, 4-EB-P1 and p70S6K phosphorylation, and (C) promoted phospho-Beclin-1 and LC3B II accumulation. Data are from >3 independent Western blots normalized by β-actin levels; *p <0.05, **p < 0.01 compared to controls. Figure S8. Fucoidan up-regulates phosphor-Akt. (A) Fucoidan increased Akt phosphorylation at Ser473 in MCF-7 cells in a concentration-dependent manner; no change in Akt phosphorylation at Thr308. (B) Fucoidan increased Akt phosphorylation at Ser473 in MDA-MB-231 cells in a concentration- and time- [file 12906_2016_1129_MOESM1_ESM.pdf]

Table S1. Panels of 248 genes analyzed for expression profiling with Nanostring™ nCounter® and the 6 housekeeping reference genes used for normalization.

Housekeeping	Cancer		Immunity			Endocrine		Kinome	Transcription	Metabolism	Structural
B-Tubulin	BECN1	IFNA1	AHR	CTF1	SOCS3	AR	LEPR	IFNK	ARNT	ACPP	COL12A1
G6DP	BIRC2	IFNGR1	ATG10	FADD	SOCS4	AGT	MSMB	IGF1R	AZGP1	AKR1C4	KRT1
GAPDH	BIRC5	IFNGR2	ATG12	IFNG	SOCS5	AGTR1	PGR	IRAK1	BAG3	AKR1D1	KRT10
POLR2A	BIRC6	IGF1	ATG5	IKBKB	SOCS7	CLCF1	PRL	IRAK2	BAG4	ANPEP	KRT14
RPL19	BIRC8	IGFBP2	ATG7	IKBKE	STAT1	CNTF	PRLR	IRAK3	ELF5	APAF1	KRT2
TBP	BRAF	JUN	BAX	IKBKG	STAT2	COMT	PROCR	IRAK4	FUR	BAK1	KRT9
	BRCA1	KLK5	BCL10	IRF1	STAT3	CUBN	SRD5A1	JAK1	HMGB2	GAA	LC3
	BRCA2	LCN2	BCL2	IRF2	STAT4	CYP11A1	SRD5A2	JAK2	KLF5	LMAN2	LMNA
	CBL	MCL1	BCL2A1	IRF3	STAT5A	CYP11B1	SULT1E1	JAK3	P56	NAT1	LMNB1
	CBLB	MED1	BCL2L11	IRF4	STAT5B	CYP11B2	SULT2A1	MAP2K2	RAD1	NAT2	SEMG1
	CBLC	MKI67	BCLAF1	IRF5	STAT6	CYP17A1	SULT2B1	MAP2K7	RAD17	PFKM	SEMG2
	CDH1	MMP3	BID	IRF6	TNFAIP2	CYP19A1	VEGF	MAP3K14	RAD9A	SERPINA3	SMOC1 (OAS)
	EGF	MMP9	BIK	IRF7	TNFRSF1A	CYP21A2	VEGFR	MAPK10	TOP2B	SMYD2	SYNE2
	EGFR	MUC1	CARD6	IRF9	TNFRSF1B	CYP7B1		NTRK1		SOS1	TP53BP2
	ENO1	P21	CASP1	ITGA6	TNFRSF21	GABARAP		PIK3R4		SOS2	
	ESR1	PPARGC1A	CASP10	ITGB4	TNFRSF25	GH2		PKA		TF	
	ESR2	PRRC2A	CASP14	JMJD6	TNFRSF9	GHR		PRKAA1			
	FANCA	PTPN11	CASP2	LGALS3BP	TNFSF10	HSD11B1		PRKACA			
	FANCC	PTPN6	CASP3	LTBR	TNFSF8	HSD11B2		PRKACB			
	FANCD2	SPRY1	CASP4	MASP2	TRAF1	HSD17B1		PRKACG			
	FANCE	SPRY2	CASP5	NFKB2	TRAF3	HSD17B2		PRKAR1A			
	FANCF	SPRY3	CASP6	NFKBIE	TRAF6	HSD17B3		PRKAR2A			
	FANCG	SPRY4	CASP7	PIGR		HSD17B7		PRKAR2B			
	FBN1	TGFa	CASP8	PRF1		HSD17B8		PTK2B			
	FOS	TNF	CASP9	RNASE2		HSD3B1		SPRED1			
	GATA	XIAP	CD70	SLC2A1		HSD3B2		SPRED2			
	GSDMA	YAP	CFHR1	SOCS1		INS		ULK1			
	HIF1A		CISH	SOCS2		LEP					

Fig. S1. Assays of toxicity. (A) Effects of FVE on membrane permeability and on mitochondrial ATP. (B) Digitonin was used as a positive control for primary necrosis. (C) Carbonyl cyanide m-chlorophenyl hydrazone (CCCP) was used as the positive control for mitochondrial toxicity.

Fig. S2. Morphological alterations. (A) FVE-untreated and (B) cells treated with 1.0 % FVE for 48 hr .

Fig. S3. Heatmap of differential mRNA expression following FVE treatment at 0.25 % and 1.0 % for 4 hr in MCF7, T47D, MDA-MB-231, HEC-1-B, RL95-2 and OVCAR-3 cell lines. Expression levels are scaled for each gene compared to untreated controls.

Significance level, $p < 0.05$.

Fig. S4. Treatment of MCF7, MDA-MB-231, HEC-1-B, MES-SA, AN3-CA, OVCAR-3 and Caov-3 cells with apoptosis (VAD) and autophagy (3MA) inhibitors.

* Indicates significant difference with FVE without inhibitor ($p<0.05$).

Fig. S5. FVE-induced apoptosis via caspase3/7-mediated PARP cleavage in MDA-MB-231 cells. Significant accumulation of the large fragment of PARP1 produced by caspase-3 cleavage was detected in MDA-MB-231 cells. The antibody does not recognize full length PARP1 or other PARP isoforms. *$p < 0.05$; **$p < 0.01$ compared to control.

Fig. S6. FVE down-regulates PI3K/Akt/mTOR signaling in MCF-7 cells. (A) FVE reduced Akt phosphorylation at Ser473 and Thr308, (B) decreased PI3K, 4-EB-P1 and p70S6K phosphorylation, and (C) promoted accumulation of phospho-Beclin 1 and LC3B II. Data are from three or more independent Western blots normalized by β-actin levels to account for loading variability. *$p < 0.05$; **$p < 0.01$ compared to control.

Fig. S7. FVE down-regulates PI3K/Akt/mTOR signaling in MDA-MB-231 cells. (A) FVE reduced Akt phosphorylation at Ser473 and Thr308, (B) decreased PI3K, 4-EB-P1 and p70S6K phosphorylation, and (C) promoted accumulation of phospho-Beclin 1 and LC3B II. Data are from three or more independent Western blots normalized by β-actin levels to account for loading variability. *$p < 0.05$; **$p < 0.01$ compared to control.

Fig. S8. Fucoidan up-regulates phosphor-Akt. (A) Fucoidan increased Akt phosphorylation at Ser473 in MCF-7 cells in a concentration dependent manner and there is no change of Akt phosphorylation at Thr308. (B) Fucoidan increased Akt phosphorylation at Ser473 in MDA-MB-231 cells in a concentration and time dependent manner and no apparent changes of Akt phosphorylation at Thr308.

Fig. S1

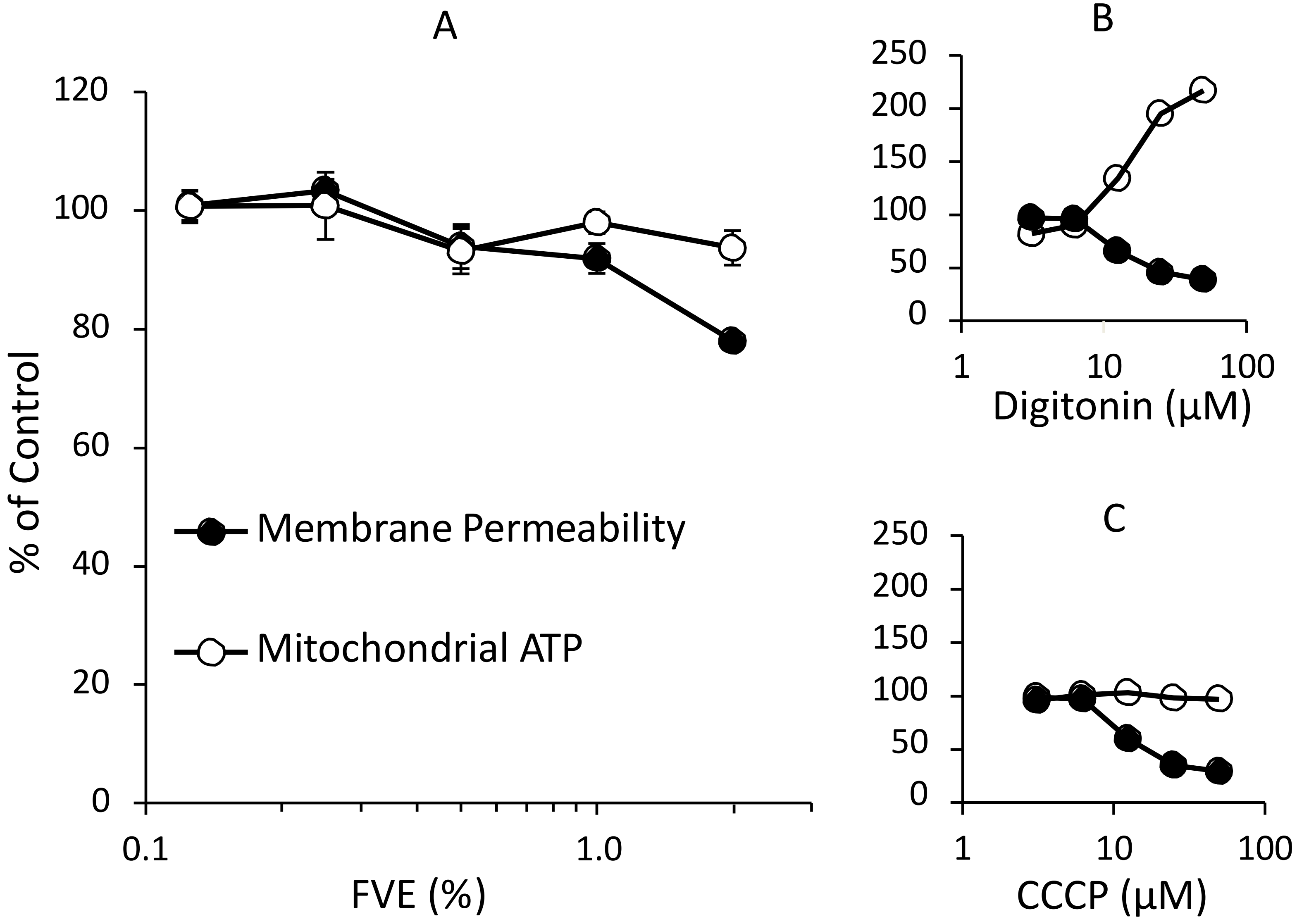
A
B
C
% of Control
120
100
80
60
40
20
0
0.1
1.0
FVE (%)
Membrane Permeability
Mitochondrial ATP
250
200
150
100
50
0
1
10
100
Digitonin (µM)
CCCP (µM)

Fig. S2

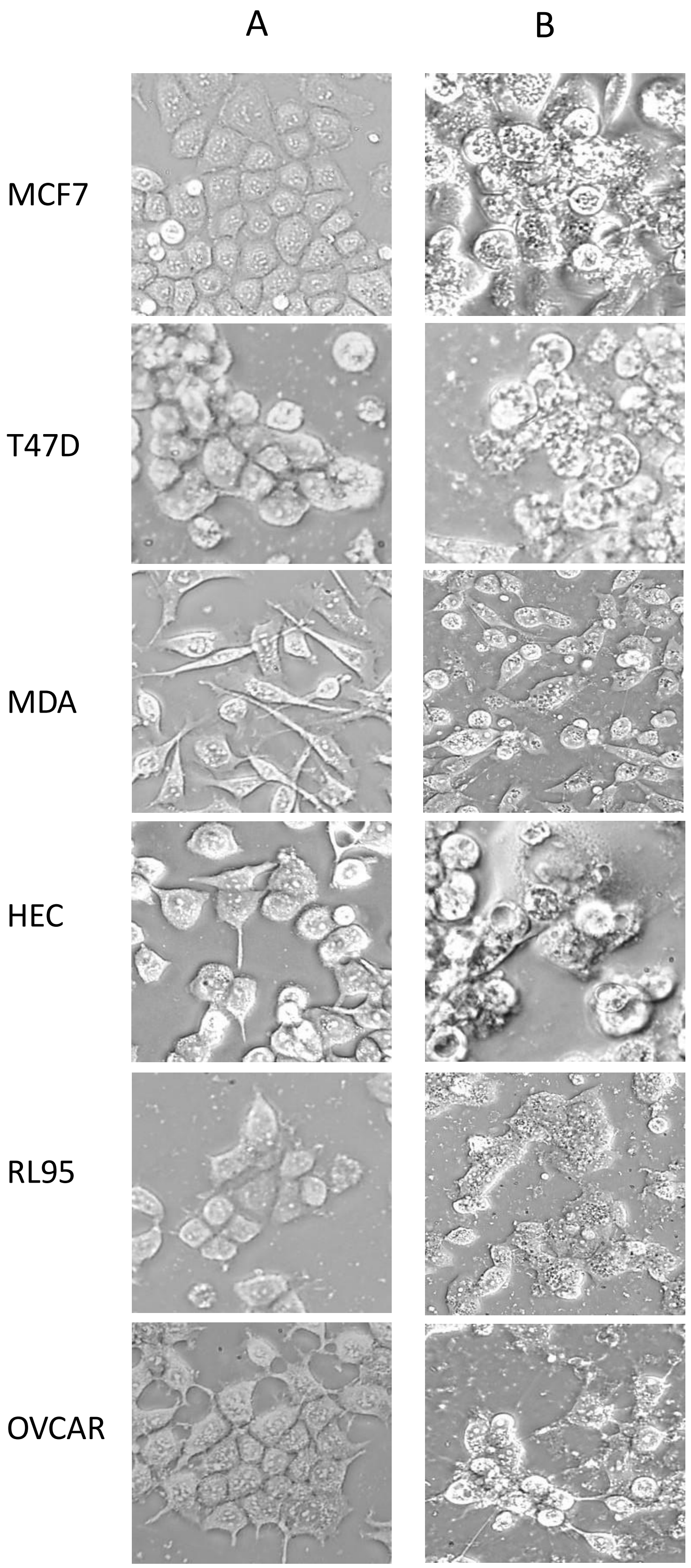

Fig. S3

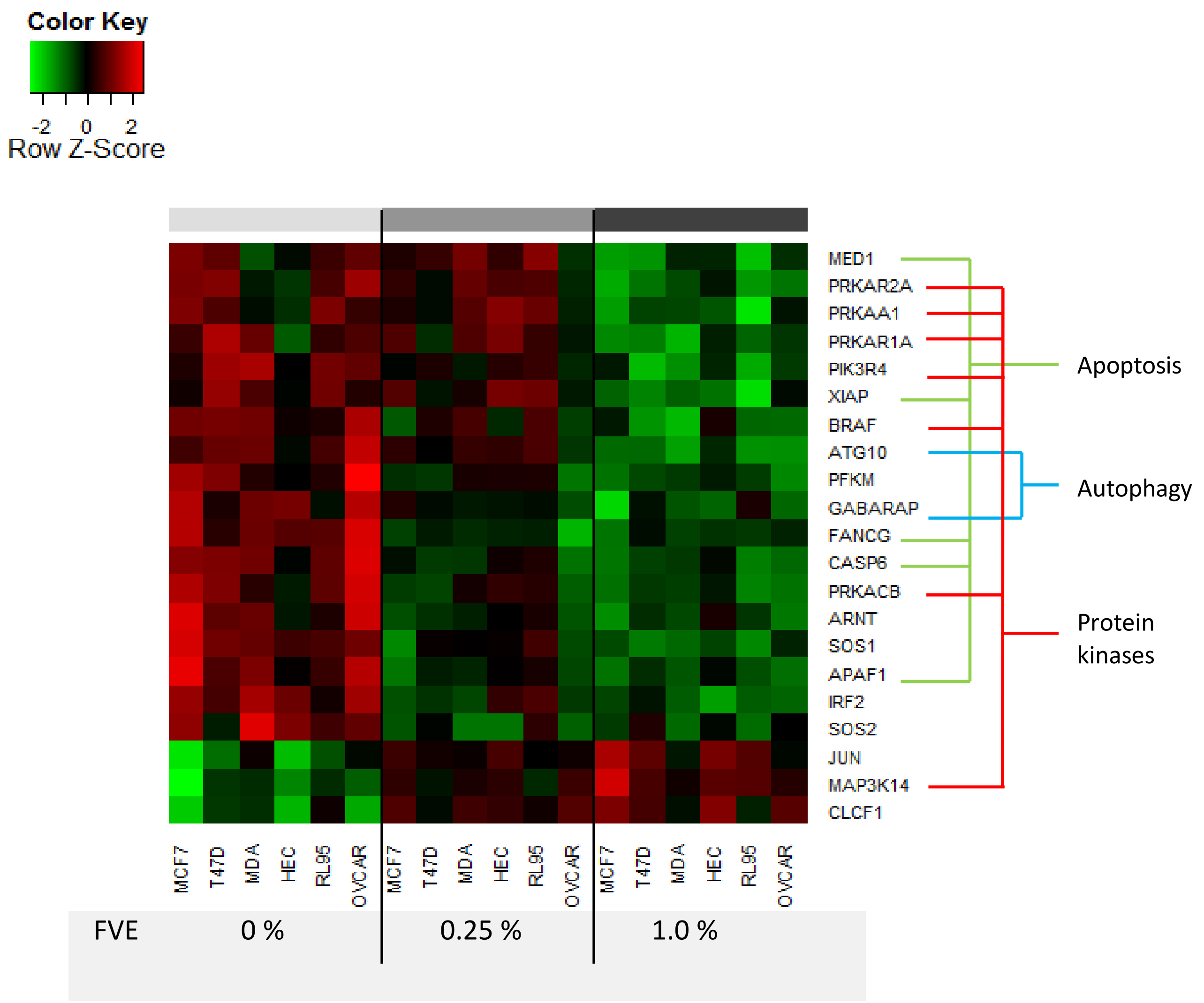

Color Key
-2
0
2
Row Z-Score
MED1
PRKAR2A
PRKAA1
PRKAR1A
PIK3R4
XIAP
BRAF
ATG10
PFKM
GABARAP
FANCG
CASP6
PRKACB
ARNT
SOS1
APAF1
IRF2
SOS2
JUN
MAP3K14
CLCF1
Apoptosis
Autophagy
Protein kinases
MCF7
T47D
MDA
HEC
RL95
OVCAR
MCF7
T47D
MDA
HEC
RL95
OVCAR
MCF7
T47D
MDA
HEC
RL95
OVCAR
FVE
0 %
0.25 %
1.0 %

Fig. S4

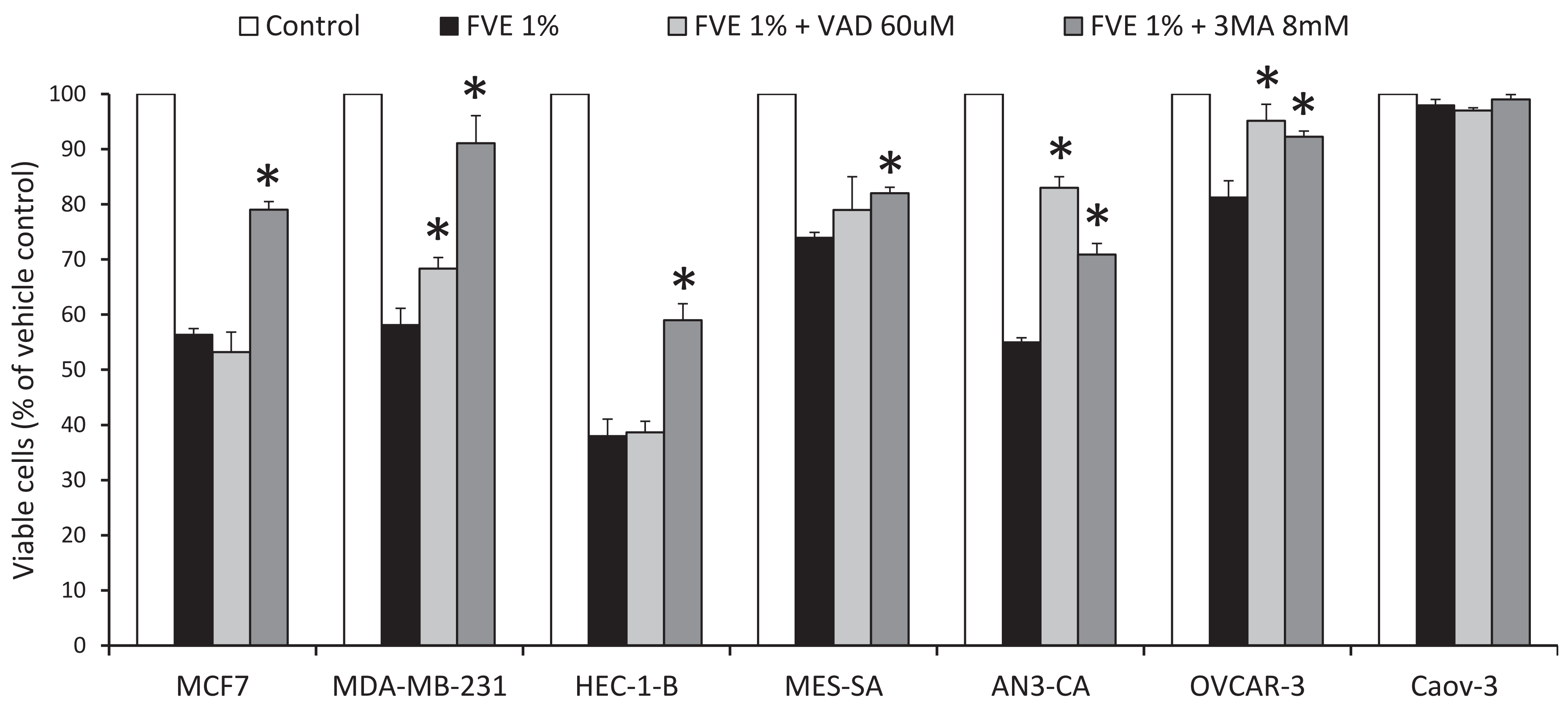

Control
FVE 1%
FVE 1% + VAD 60uM
FVE 1% + 3MA 8mM
Viable cells (% of vehicle control)
100
90
80
70
60
50
40
30
20
10
0
MCF7
MDA-MB-231
HEC-1-B
MES-SA
AN3-CA
OVCAR-3
Caov-3

Fig.S5

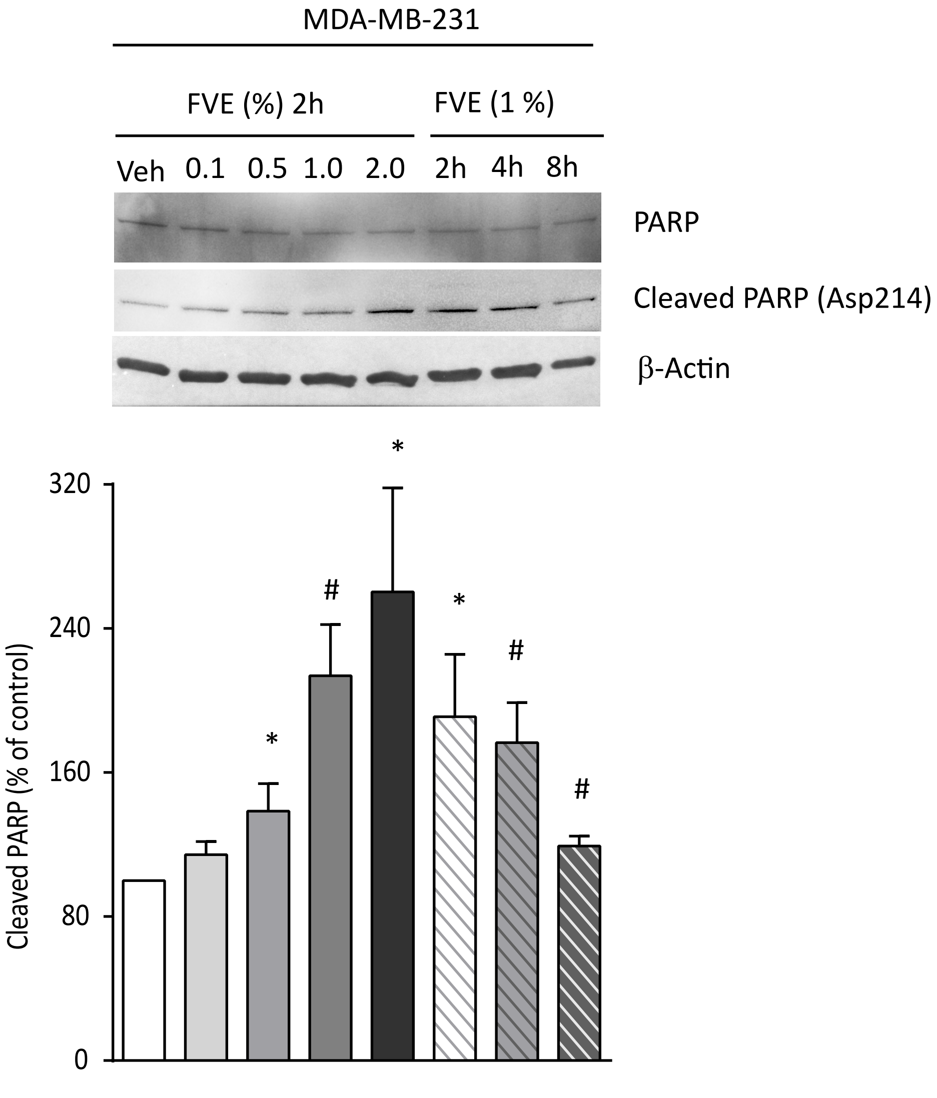

MDA-MB-231
FVE (%) 2h
FVE (1 %)
Veh
0.1
0.5
1.0
2.0
2h
4h
8h
PARP
Cleaved PARP (Asp214)
β-Actin
Cleaved PARP (% of control)
320
240
160
80
0
*
#
*
*
#
#

Fig. S6

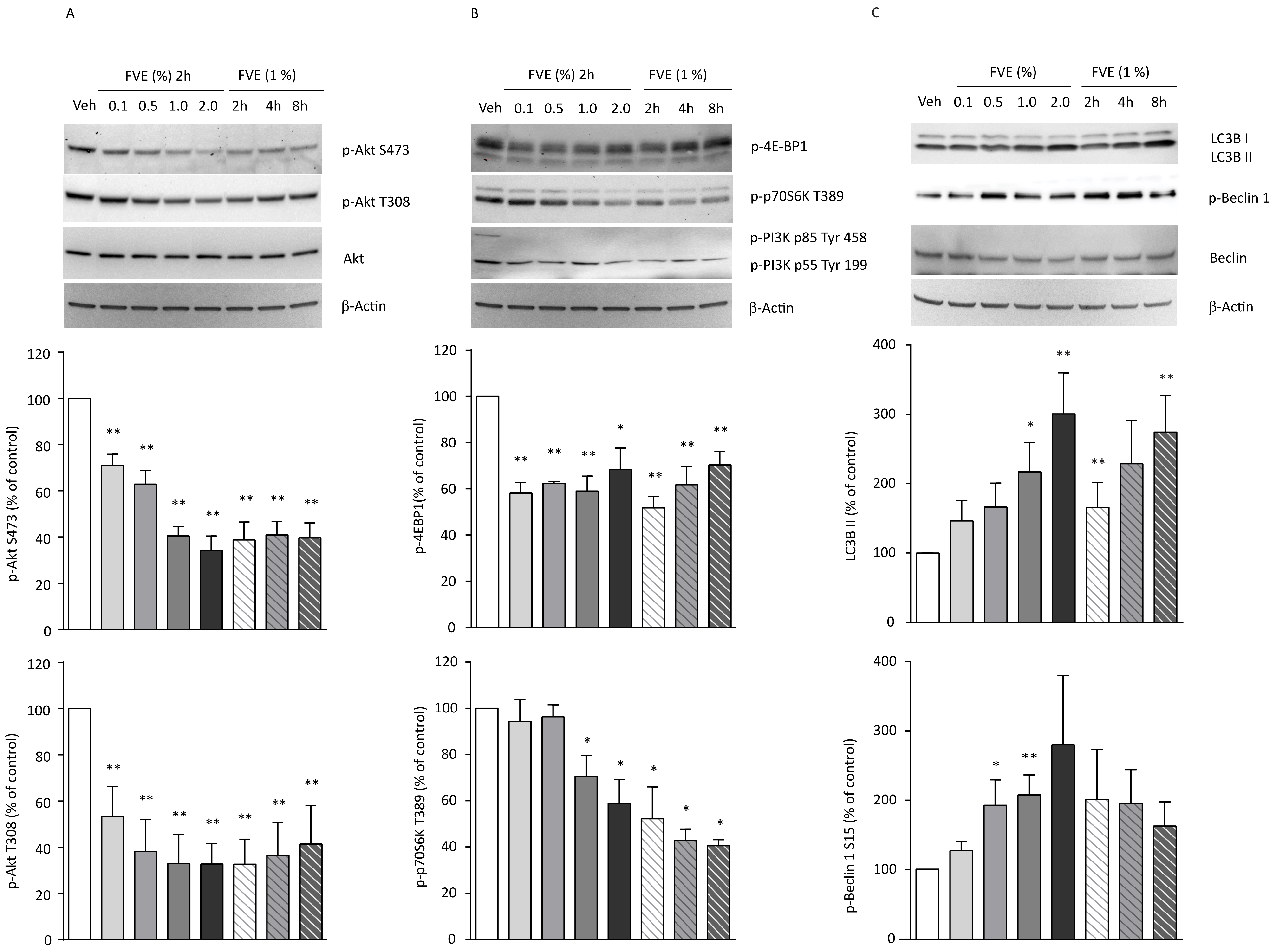
A
FVE (%) 2h
FVE (1 %)
Veh
0.1
0.5
1.0
2.0
2h
4h
8h
p-Akt S473
p-Akt T308
Akt
β-Actin
p-Akt S473 (% of control)
p-Akt T308 (% of control)
B
p-4E-BP1
p-p70S6K T389
p-PI3K p85 Tyr 458
p-PI3K p55 Tyr 199
β-Actin
p-4EBP1(% of control)
p-p70S6K T389 (% of control)
C
FVE (%)
LC3B I
LC3B II
p-Beclin 1
Beclin
β-Actin
LC3B II (% of control)
p-Beclin 1 S15 (% of control)

Fig. S7

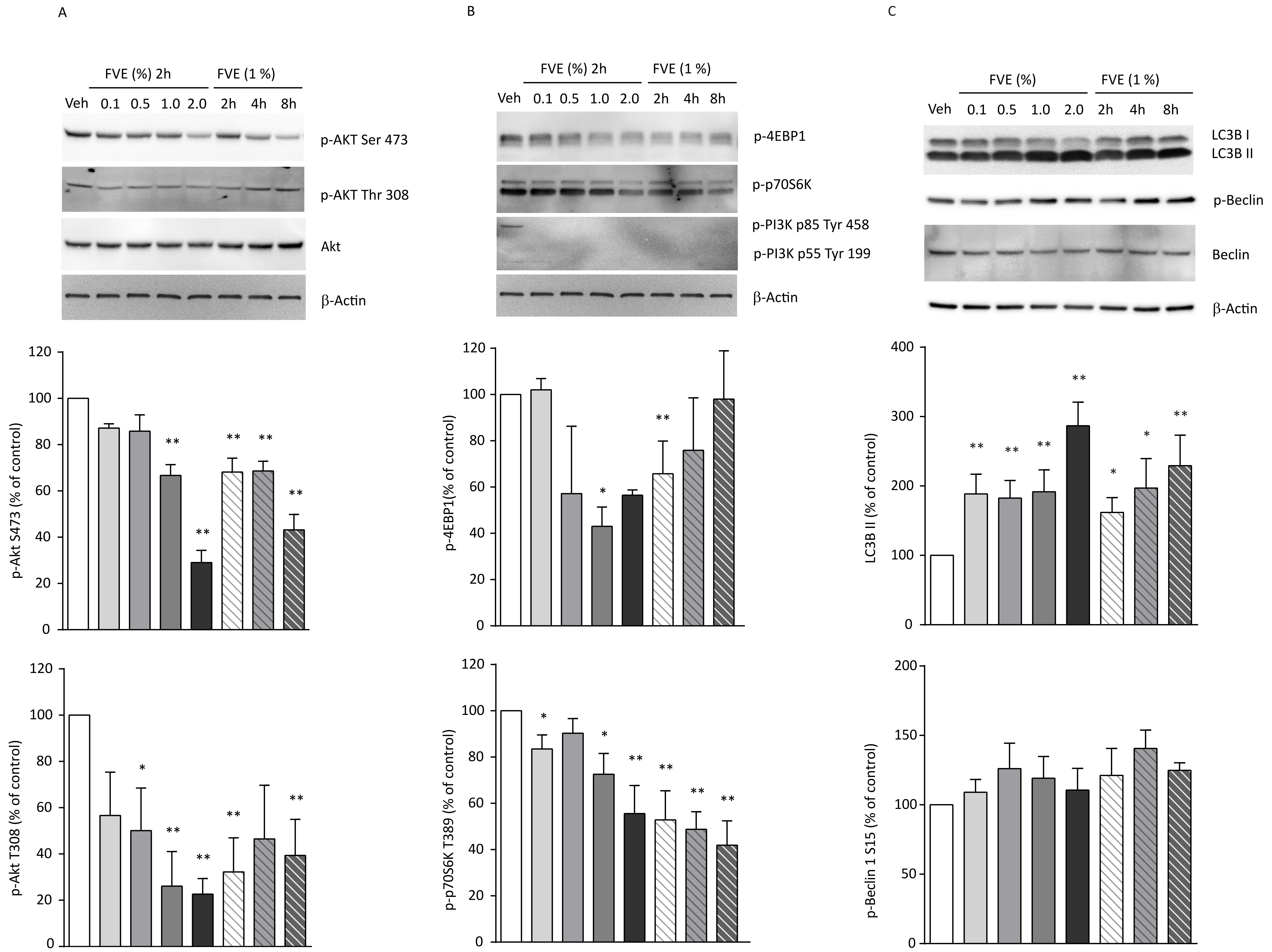
A
FVE (%) 2h
FVE (1 %)
Veh
0.1
0.5
1.0
2.0
2h
4h
8h
p-AKT Ser 473
p-AKT Thr 308
Akt
β-Actin
p-Akt S473 (% of control)
p-Akt T308 (% of control)
B
p-4EBP1
p-p70S6K
p-PI3K p85 Tyr 458
p-PI3K p55 Tyr 199
p-4EBP1(% of control)
p-p70S6K T389 (% of control)
C
FVE (%)
LC3B I
LC3B II
p-Beclin
Beclin
LC3B II (% of control)
p-Beclin 1 S15 (% of control)

Fig. S8

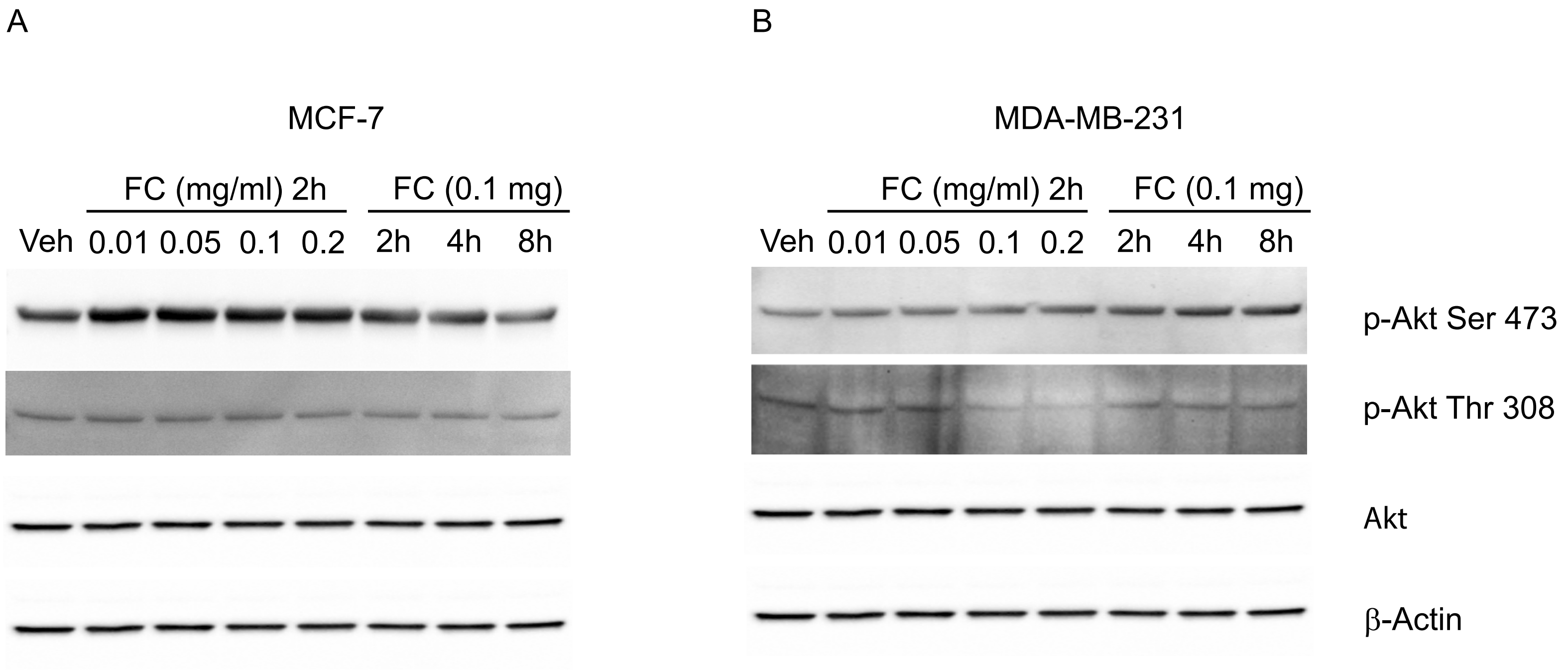
A
MCF-7
FC (mg/ml) 2h
FC (0.1 mg)
Veh 0.01 0.05 0.1 0.2 2h 4h 8h
B
MDA-MB-231
FC (mg/ml) 2h
FC (0.1 mg)
Veh 0.01 0.05 0.1 0.2 2h 4h 8h
p-Akt Ser 473
p-Akt Thr 308
Akt
β-Actin
